# Biochemical characterization of malate synthase G of *P. aeruginosa*

**DOI:** 10.1186/1471-2091-10-20

**Published:** 2009-06-24

**Authors:** Bart Roucourt, Nikki Minnebo, Patrick Augustijns, Kirsten Hertveldt, Guido Volckaert, Rob Lavigne

**Affiliations:** 1 Division of Gene Technology, Department of Biosystems, Katholieke Universiteit Leuven, Kasteelpark Arenberg 21, box 2462, Leuven, BE-3001, Belgium; 2 Laboratory for Pharmacotechnology and Biopharmacy, Katholieke Universiteit Leuven, Campus Gasthuisberg O&N 2, Box 921, Herestraat 49, 3000 Leuven, Belgium

## Abstract

**Background:**

Malate synthase catalyzes the second step of the glyoxylate bypass, the condensation of acetyl coenzyme A and glyoxylate to form malate and coenzyme A (CoA). In several microorganisms, the glyoxylate bypass is of general importance to microbial pathogenesis. The predicted malate synthase G of *Pseudomonas aeruginosa *has also been implicated in virulence of this opportunistic pathogen.

**Results:**

Here, we report the verification of the malate synthase activity of this predicted protein and its recombinant production in *E. coli*, purification and biochemical characterization. The malate synthase G of *P. aeruginosa *PAO1 has a temperature and pH optimum of 37.5°C and 8.5, respectively. Although displaying normal thermal stability, the enzyme was stable up to incubation at pH 11. The following kinetic parameters of *P. aeruginosa *PAO1 malate synthase G were obtained: K_m glyoxylate _(70 μM), K_m acetyl CoA _(12 μM) and V_max _(16.5 μmol/minutes/mg enzyme). In addition, deletion of the corresponding gene showed that it is a prerequisite for growth on acetate as sole carbon source.

**Conclusion:**

The implication of the glyoxylate bypass in the pathology of various microorganisms makes malate synthase G an attractive new target for antibacterial therapy. The purification procedure and biochemical characterization assist in the development of antibacterial components directed against this target in *P. aeruginosa*.

## Background

Malate synthase catalyzes the condensation of acetyl coenzyme A and glyoxylate to form malate and coenzyme A (CoA) [1]. Along with isocitrate lyase, which catalyses the aldol cleavage of isocitrate to succinate and glyoxylate, this enzyme constitutes the glyoxylate bypass of the tricarboxylic acid (TCA) cycle [2]. Apart from replenishing the TCA cycle intermediates (anaplerotic role), the glyoxylate cycle is essential for growth on acetyl CoA (from fatty acids or from acetate) as a sole carbon source by bypassing two decarboxylation steps of the TCA cycle. Therefore, the carbon imported in the glyoxylate cycle as acetyl is not lost as CO_2 _– as would happen in the TCA cycle – but incorporated into intermediates of the cycle, which are available for anabolism. The net result is the conversion of two molecules acetyl CoA into one molecule of succinate. Because the glyoxylate cycle includes two oxidative steps, it also provides some energy [2].

Recently, Fischer and Sauer reported that malate synthase is an important component of the phosphoenolpyruvate-glyoxylate cycle in *Escherichia coli*, which functions under conditions of glucose hunger [3]. In addition, it serves an anaplerotic role in glycolate oxidation [4,5]. In several microorganisms, the glyoxylate bypass is of general importance to microbial pathogenesis. Persistence of *Mycobacterium tuberculosis *is facilitated by the glyoxylate bypass [6], whereas this metabolic pathway is required for high virulence of *Candida albicans *[7]. The ability of these organisms to utilize fatty acids as a carbon and energy source seems crucial to their survival in the infected host. Although it is widely accepted that the glyoxylate cycle operates in bacteria, fungi, some protists, and plants, the claim that the glyoxylate cycle is functionally active in higher animals remains controversial [8]. In *E. coli *two isoenzymes have been identified: *aceB *encodes the malate synthase A involved in acetate metabolism whereas *glcB *encodes the malate synthase G (MSG) involved in glycolate metabolism [9,10]. *Pseudomonas aeruginosa *is predicted to encode MSG (*glcB *or PA0482 gene) but not type A [11].

The expression of *glcB *of *P. aeruginosa *is upregulated during infection [12] and the malate synthase activity is implicated in twitching-mediated chemotaxis towards phospholipids and fatty acids [13]. Both elements suggest the importance of the glyoxylate bypass in the pathogenesis of *P. aeruginosa*. In view of possible applications in the development of novel antibiotics against *P. aeruginosa*, we have characterized the MSG of this opportunistic pathogen endowed with strong antibiotic resistance [14,15].

## Results

### Deletion of the *P. aeruginosa *gene encoding MSG

**Figure 1:**
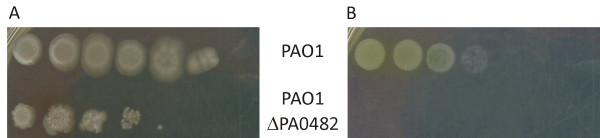
**Inability of the MSG deletion mutant to grow on acetate as sole carbon source**. *P. aeruginosa *PAO1 wild type (PAO1) and the MSG deletion mutant (PAO1 ΔPA0482) were spotted on (A) LB and (B) minimal medium with acetate as sole carbon source in a tenfold dilution series.

The *glcB *gene of *P. aeruginosa *PAO1 encoding MSG was deleted by double homologous recombination. *P. aeruginosa *PAO1 Δ*glcB *is able to grow on rich medium (LB medium), although with slightly reduced efficiency of plating, compared to the wild type strain (Figure 1). The deletion mutant was unable to produce colonies on medium with acetate as the sole carbon source, confirming the essential nature of the malate synthase for growth on acetate and the prediction of a single MSG gene (Figure 1). The reduced efficiency of plating of wild type *P. aeruginosa *and the reduced colony size on media with acetate indicate suboptimal growth of wild type *P. aeruginosa *on acetate.

### Recombinant production and purification of MSG

**Figure 2:**
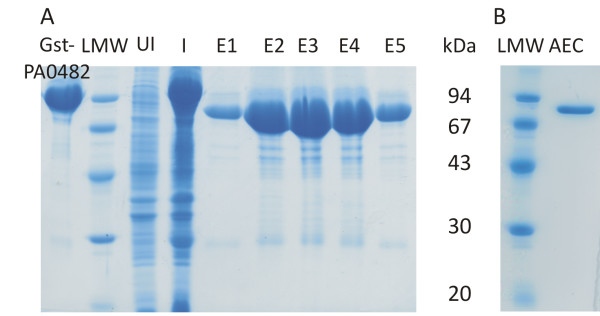
**Recombinant production and purification of the malate synthase G of *P. aeruginosa *PAO1**. (A) purification of malate synthase G after removal of the GST tag is shown and (B) after anion exchange chromatography (AEC). Samples from purified Gst-malate synthase fusion protein (Gst-PA0482), samples from the uninduced (UI) and induced (I) cultures are compared to the elution fractions (E1 to 5) after glutathione purification with removal of the GST tag and anion exchange chromatography. The amounts are not proportional between A and B. The low molecular weight ladder (LMW) served as reference for both gels (the molecular weight of the markers is indicated in kDa).

The MSG was produced recombinantly fused to a glutathione S-transferase (GST) tag. The protein was purified to homogeneity by subsequent glutathione affinity chromatography, proteolytic removal of the GST tag and anion exchange chromatography. Approximately 45 mg of MSG was purified per liter of culture broth. The predicted molecular weight of the fusion protein (959 amino acids, 105.9 kDa) as well as MSG (733 amino acids, 79.4 kDa) corresponds well to the observed bands on gel (Figure 2).

### Influence of temperature on the activity and stability of MSG

**Figure 3:**
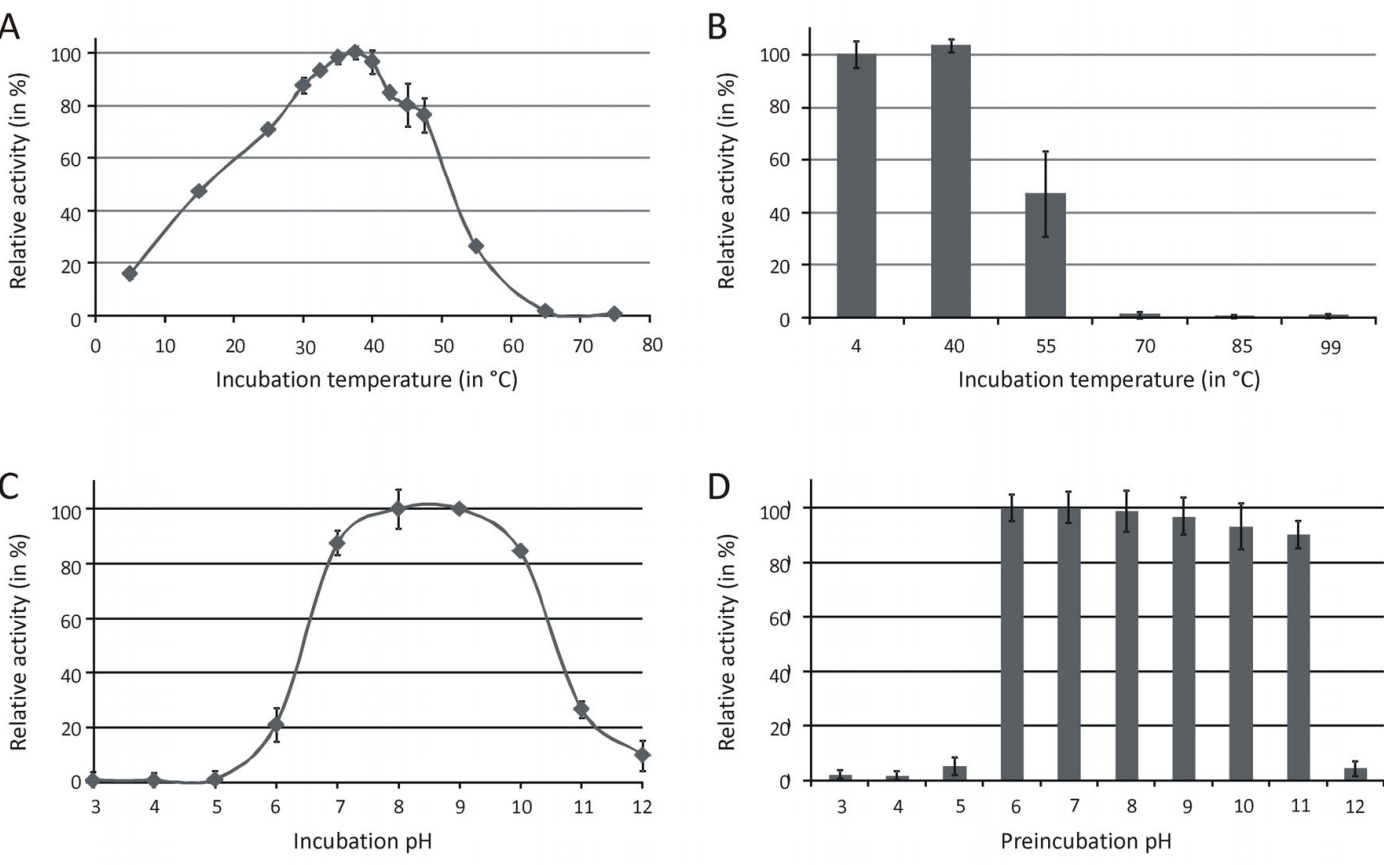
**Influence of the temperature and pH on MSG activity**. The malate synthase activity was expressed relative to the activity at 37.5°C or pH8 for (A) the temperature and (C) pH optimum, respectively. (B) Temperature and (D) pH stability were expressed relative to the activity after a preincubation at a temperature of 4°C or pH 7, respectively.

Both the temperature optimum and the thermal stability of the MSG activity were investigated. To achieve a higher resolution, the zone of highest activity was measured at smaller temperature intervals (2.5°C). Maximal activity occurs at 37.5°C (Figure 3A); between 32.5 and 40°C more than 90% of the activity remains. At lower temperatures, this activity decreases steadily, but does not drop to zero. At 5°C and 15°C, 15.5% and 47.1% of the activity remained, respectively. Above 47.5°C the activity dropped fast and was not significantly different from zero at 65°C and 75°C (two tailed t-test, P = 0.99) (Figure 3A).

The thermal stability of MSG was examined by preincubating the enzyme for one hour at a given temperature and subsequently assaying the enzymatic activity at 37.5°C as described in the previous paragraph. Preincubation of MSG at 40°C or lower temperatures did not result in reduced enzymatic activity (Figure 3B). After one hour preincubation at 55°C, enzymatic activity was approximately halved (53% drop). Higher preincubation temperatures (70°C, 85°C and 99°C) caused complete loss of malate synthase activity (Figure 3B).

### Influence of pH on the activity and stability of MSG

The pH optimum of MSG was investigated using standard conditions (an incubation temperature of 37°C). To eliminate a possible influence of the buffering components on the enzymatic activity, a universal buffer containing three different buffering agents (citrate, Tris and borate) was used. The MSG activity is maximal between pH 8 and 9 (Figure 3C). At a pH of 7 and 10, 87 and 85% of the activity is retained, respectively. However, outside this pH range (below pH 7 and above pH 10) the activity decreases fast. Below pH 6 no activity remains (Figure 3C).

The pH dependency of MSG stability was determined by preincubation of the enzyme for one hour at a specific pH and subsequently diluting the enzyme 100-fold before assaying the enzymatic activity at pH 7.5. Stability of MSG was maximal after a preincubation at pH 6 and 7 (Figure 3D). When preincubating the enzyme in more basic buffer (pH 8 to pH 11), the stability gradually dropped (at pH 11, still 90% of the activity was retained). The enzymatic activity was lost upon preincubation at lower (≤ pH 5) or higher pH values (≥ pH 12) (Figure 3D).

### MSG is susceptible to inhibition by pyruvate

**Figure 4:**
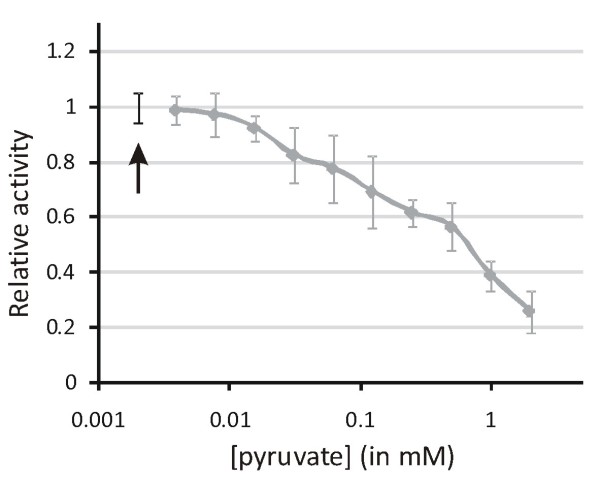
**The influence of pyruvate on the malate synthase activity**. The relative activity of malate synthase G is shown in function of the pyruvate concentration (in mM). The reaction without pyruvate (indicated by the black arrow) serves as reference.

The inhibitory potential of pyruvate on the activity of the MSG of *P. aeruginosa *was shown by adding a twofold dilution series of pyruvate to a constant amount of MSG and a constant amount of substrate. The malate synthase activity dropped with increasing pyruvate concentration (Figure 4), showing that pyruvate also acts as an inhibitor of the *P. aeruginosa *MSG.

### Kinetic parameters of MSG

**Figure 5:**
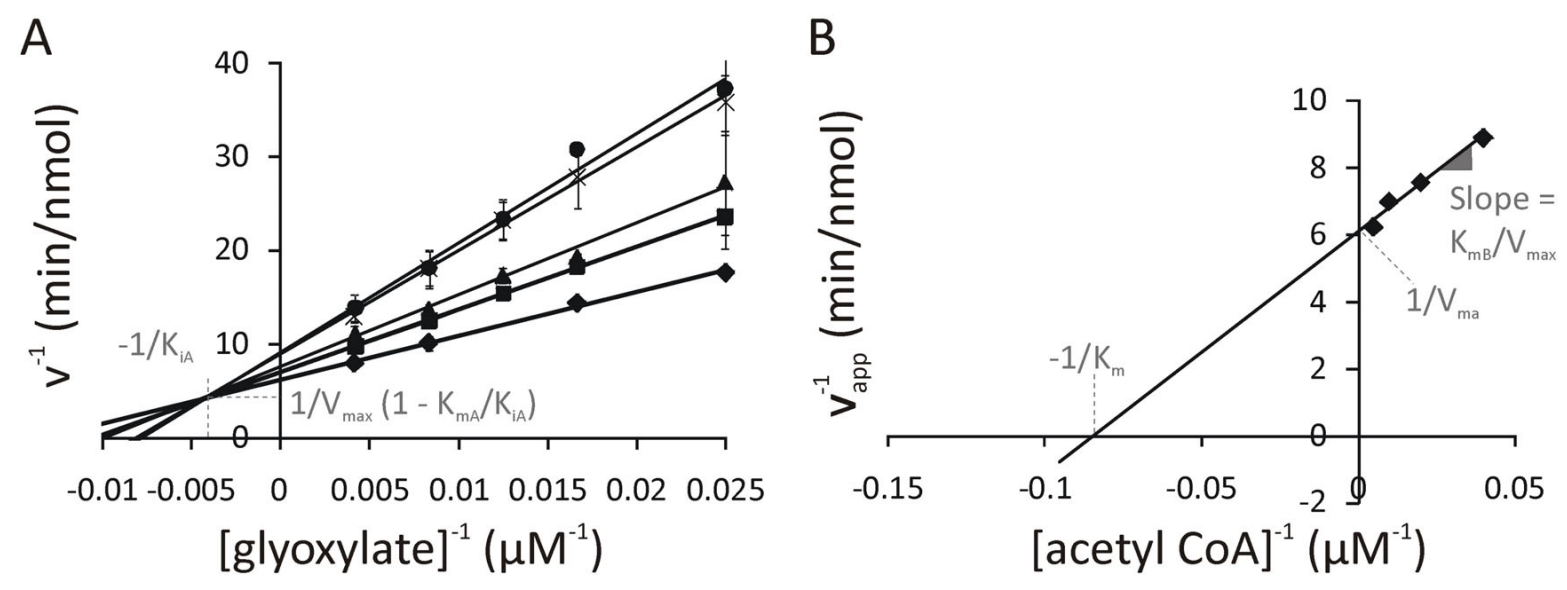
**Determination of the kinetic parameters of malate synthase G of *P. aeruginosa***. The formulas for the calculation of the kinetic parameters are indicated. In the formulas, subscript A and B refer to glyoxylate and acetyl CoA, respectively. (A) The primary Lineweaver-Burk plots show reciprocal velocity (minutes/nmol) in function of the reciprocal glyoxylate concentration (in μM^-1^). Each line represents a concentration of acetyl CoA: 200 (black diamond), 100 (black square), 50 (black triangle), 25 (black X), 15 μM (black dot). R^2 ^of the trendlines are 0.995, 0.999, 0.989, 0.996 and 0.982, respectively. (B) The secondary plot gives the reciprocal apparent velocities (in minutes/nmol) as a function of the reciprocal acetyl CoA concentration (in μM^-1^). The R^2 ^of the trendline is 0.9781.

To determine the kinetic parameters of MSG, the enzymatic reaction was followed over time. Different combinations of substrate (acetyl CoA and gloxylate) concentrations were assayed, whereas the other conditions remained constant (pH 7.5, 25°C, the amount of MSG). For each combination, the velocities were determined. The MSG-catalyzed reaction generated linear Lineweaver-Burk plots over the concentration range tested. Figure 5A shows these plots with respect to glyoxylate at various concentrations of acetyl CoA. Since this is a two-substrate reaction, determination of the actual parameters requires a secondary plot (Figure 5B). The formulas indicated in the figures allow calculation of kinetic parameters. The K_m glyoxylate_(70 μM), K_m acetyl CoA _(12 μM) and V_max _(16.5 μmol/minutes/mg MSG) of the *P. aeruginosa *PAO1 MSG are comparable to those of other malate synthases available from literature (Table 1, [16-21]).

**Table 1:** Comparison of the kinetic parameters of malate synthases of the organisms listed.

Organism	Type	K_m glyoxylaat _(μM)	K_m acetyl-CoA _(μM)	V_max _(μmol/min/mg)	Reference
***P. aeruginosa***	**MSG**	**70**	**12**	**16.5**	**This work**
*M. tuberculosis*	MSG	57	30	6	[16]
*E. coli*	MSG	25	9	36	[17]
*Streptomyces coelicolor*	MSA	3,49	0,035	26.2	[18]
*S. clavuligerus*	MSA	0,59	0,029	11.8	[18]
*Corynebacterium glutamicum*	MSA	30	12	Unknown	[19]
*Saccharomyces cerevisiae*	MSA	100	83	Unknown	[20]
*Zea mays*	MSA	104	20	Unknown	[21]

## Discussion

Malate synthase is essential for the growth on acetate as a sole carbon source, which is in agreement with the prediction of a single malate synthase in *P. aeruginosa*. In addition, the deletion mutant shows somewhat reduced growth on rich medium, probably reflecting its anaplerotic role in the TCA cycle and its role in the phosphoenolpyruvate-glyoxylate cycle, as described by Fischer and Sauer [3]. An efficient and straightforward procedure for the purification of untagged MSG was developed. Removal of the GST tag rules out possible interference of the tag with the enzymatic activity of the enzyme. In addition, the quantity and the purity of the tagless enzyme are suitable for crystallography of *P. aeruginosa *MSG.

Subsequently, the purified MSG activity was characterized biochemically. The temperature optimum and stability show that the enzyme behaves like a typical mesophylic protein, since these parameters indicate a rapid and irreversible inactivation of MSG. The reduction in thermal stability from 55°C on is in agreement with the steep decline in enzymatic activity at this temperature. After all, instability decreases the amount of functional enzyme. However, reduction in enzymatic activity at 55°C (incubation for 30 minutes, reduction of 74%) is only partially explained by the thermal instability at 55°C (incubation for one hour, reduction of 53%). This suggests that a fraction of the protein is unfolded reversibly – and therefore temporarily inactive – before either denaturing irreversibly at this temperature or returning to its active state after cooling. The temperature optimum (37.5°C) and the considerable activity at lower temperatures (at 15°C nearly half of the activity remains) are in agreement with the microbial characteristics of *P. aeruginosa *as an opportunistic (human) pathogen and a common water and soil organism, respectively. With only 90% of the activity realized at the cytoplasmic pH, the pH optimum and stability of MSG seem somewhat shifted to a more basic pH range. The stability analysis suggests fast and irreversible denaturation of MSG outside the pH range from 6 to 11. The optimal pH for enzymatic activity (above 85%) was limited to a more narrow pH range (pH 7 to 11). In combination with the slightly reduced stability at pH 9 to 11, these data suggest that the actual pH optimum for the enzymatic activity is shifted somewhat further to a more basic pH compared to the apparent pH optimum (between 8 and 9). Since the enzyme functions intracellularly (at a periplasmic pH between 7.2 and 7.4) [22], the high activity at elevated pH is quite surprising. The reason for this remains to be elucidated, as data from other malate synthases is lacking. Possibly, this pattern can be explained by the catalytic mechanism of MSG and the residues involved. Based on sequence similarity with MSG of *E. coli *[17,23] and *M. tuberculosis *[16,24], Asp^631 ^functions as a catalytic base, abstracting a proton from the terminal methyl group of acetyl CoA. The resulting negative enolate is stabilized by the positive charge of conserved Arg^340^. Glyoxylate is polarized for nucleophilic attack by the magnesium ion, which is essential for enzymatic activity. After nucleophilic attack of the enolate anion on glyoxylate, the resulting malyl-CoA intermediate is stabilized by Mg^2+ ^and Arg^340^. Subsequently, this intermediate is hydrolyzed by a water molecule activated by Glu^274 ^and/or Asp^275^. As a result, the products, malate and CoA, are released [16,17,23,24]. The theoretical pK_a_'s of the side chains of the catalytic residues (4.1 (Asp), 4.5 (Glu), 12.0 (Arg) in solution at 25°C) suggest that their charge probably remains unaltered over this pH range. However, the pK_a _can vary considerably (more than one pH unit) depending on the local environment of the amino acid side chain in the protein [25]. Alternatively, the charge of residues involved in substrate binding might change over this pH range. Likewise, charge changes of residues crucial to the structural stability of MSG could gravely affect the enzyme's structural stability at a pH outside the range between 6 and 11 and lead to (irreversible) denaturation. Future structural analysis of this MSG might provide more insight in the exact mechanisms involved. The inhibition of malate synthase G by pyruvate is probably competitive in nature, presumably by competition with glyoxylate [17].

The kinetic parameters of MSG of *P. aeruginosa *are comparable to those of the enzymes of other organisms (Table 1). Since the extended straight lines in the primary Lineweaver-Burk plot intersect approximately in one unique point, MSG proceeds through a ternary complex, which contains the enzyme in complex with both substrates, as opposed to a substituted enzyme mechanism, in which a group is transferred from the first substrate that leaves the complex before the second substrate binds. This is in agreement with the catalytic mechanism proposed for the *E. coli *[17,23] and the *M. tuberculosis *MSG [16,24] as is the case for most enzymes with two substrates catalyzing a group transfer [26].

## Conclusion

The implication of the glyoxylate bypass in the pathology of various microorganisms makes MSG an attractive new target for antibacterial therapy [6,7,12]. What makes them especially appealing is that no genes encoding the enzymes of the glyoxylate bypass have been identified in mammals [8]. In addition, *P. aeruginosa *was shown to encode only a single malate synthase. The microbial and biochemical characterization of MSG of *P. aeruginosa *are an important step towards the development of antibacterials targeting this enzyme to combat antibiotic resistant *P. aeruginosa *infections. Future studies of the inhibition kinetics of pyruvate in combination with the elucidation of the crystal structure of MSG in complex with substrates, products and natural inhibitors will be instrumental to develop novel inhibition strategies.

## Methods

### Construction of the *glcB *deletion mutant

The construction of the deletion mutant was described previously [27]. Drops (2 μl) of a tenfold dilution series of an overnight culture of wildtype *P. aeruginosa *or the *glcB *deletion mutant were spotted on LB medium and M9 minimal medium. M9 minimal medium was composed of 11.28 g/l M9 salts (Difco Laboratories, Detroit, MI, USA), 2 mM MgSO_4 _(Acros Organics, Geel, Belgium), 0.1 mM CaCl_2 _(Sigma-Aldrich), 0.5% w/v of acetate (Merck, Darmstadt, Germany) as the sole carbon source, and 1.5% w/v of agar (Lab^M^).

### Recombinant production of MSG in *E. coli*

The 2,175 bp open reading frame (ORF) encoding malate synthase G was PCR amplified (GoTaq DNA polymerase, 2.5 units, Promega, Madison, WI, USA) using the *P. aeruginosa *PAO1 genome as template and inserted in the multiple cloning site of pGEX-6P-1 (GE Healthcare, Little Chalfont, UK). DNA sequence analysis confirmed the sequence of the inserted ORF and the reading-frame fusion with the coding sequence of N-terminal GST tag. The MSG and the GST tag are separated by the recognition sequence (LEVLFQGP) of the Prescission™ protease (GE Healthcare). *E. coli *BL21 cells containing the pGEX-6P-1-*glcB *construct were grown in 2xTY to an optical density (at 600 nm) of 0.6 and induced overnight at 16°C using 0.1 mM Isopropyl β-D-1-thiogalactopyranoside (IPTG).

In a first step, the recombinant protein was purified from cleared lysates on an Δkta FPLC (GE Healthcare) using a 5 ml GSTrap HP column (GE Healthcare) as described by the manufacturer. For proteolytic removal of the GST tag, elution was performed with 50 units of Prescission™ protease (GE Healthcare) after overnight on column incubation with the protease at 4°C. The next day, the released MSG was eluted (50 mM Tris.HCl, pH 7.5). Prior to the second purification step using anion exchange chromatography (AEC), the eluate resulting from the Prescission™ protease-based release from the GSTrap HP column was dialyzed against a triethanolamine buffer (20 mM pH 7.5). The sample was loaded on Q sepharose Fast Flow column (15 × 160 mm, GE Healthcare). After washing the column (200 ml at 2 ml/min), the proteins were eluted using a linear gradient of 0 to 0.5 M NaCl in 150 minutes (1 ml/min) and collected in 2 ml fractions. The purified fractions were pooled and dialyzed against a buffer compatible with the malate synthase activity assays (100 mM Tris.HCl, 5 mM MgCl_2 _at a pH of 7.5).

### MSG activity assays

The temperature and pH optimum and stability, as well as the inhibition by pyruvate, were performed with an endpoint assay which measures the amount of CoA. The amount of free thiol groups of CoA was determined using 5, 5'-dithiobis-(2-nitrobenzoic acid) or DTNB (Sigma-Aldrich, St. Louis, MO, USA)[5]. The absorption at 415 nm was measured in a microplate reader (Microplate reader model 680, Bio-Rad Laboratories, Hercules, CA, USA). DTNB was added to a final concentration of 2 mM. Addition of DTNB to the mixture arrests the reaction, probably due to reaction of the DTNB with the free thiol groups of cysteine [21]. The standard reaction mixture contains 280 ng MSG, 100 mM Tris.HCl, 5 mM MgCl_2_, 2 mM glyoxylate (Sigma-Aldrich), 1 mM acetyl CoA (AppliChem GmbH, Darmstadt, Germany). Unless stated otherwise, the reactions were incubated for 30 min at pH 7.5. To identify the pH optimum, reactions were buffered using a universal buffer spanning a pH range from 2 to 12 (30 mM citrate, 30 mM Tris, 30 mM borate). All experiments were conducted in triplicate. The error bars correspond to one standard deviation.

To determine the kinetic parameters of MSG, the reaction was followed over time by measuring the absorption of the acetyl thioester bond at 232 nm [21]. The assays were performed in transparent microplates (Greiner, Frickenhausen, Germany), incubated at 25°C and measured with a TECAN Infinite^® ^M200 (TECAN, Zurich, Switzerland). Each reaction contained 10 μg of enzyme. All combinations of 15, 25, 50, 100 and 200 μM acetyl CoA with 40, 60, 80, 120 and 240 μM glyoxylate were assayed at 25°C and pH 7.5 in triplicate. The error bars correspond to one standard deviation. Kinetic parameters (K_m _and V_max_) were calculated from Lineweaver-Burk plots as described by Cornish-Bowden [26].

## Authors' contributions

BR, GV and RL designed the concept and experiments of this study. BR, NM were involved in the acquisition of the data. In addition to BR and NM, PA was specifically involved in data acquisition for determination of the kinetic parameters. BR, NM, KH and RL were involved in the analysis and interpretation of the data. BR drafted the manuscript, whereas the other authors helped to draft the manuscript. All authors have approved the final manuscript.
